# A Brief Review of Poly(Vinyl Alcohol)-Based Anion Exchange Membranes for Alkaline Fuel Cells

**DOI:** 10.3390/polym14173565

**Published:** 2022-08-29

**Authors:** Asep Muhamad Samsudin, Merit Bodner, Viktor Hacker

**Affiliations:** 1 Institute of Chemical Engineering and Environmental Technology, Graz University of Technology, 8010 Graz, Austria; 2 Department of Chemical Engineering, Diponegoro University, Semarang 50275, Indonesia

**Keywords:** alkaline fuel cell, anion exchange membrane, fuel cell, poly(vinyl alcohol), preparation, hydroxide conductivity, fuel permeability

## Abstract

Anion exchange membrane fuel cells have unique advantages and are thus gaining increasing attention. Poly(vinyl alcohol) (PVA) is one of the potential polymers for the development of anion exchange membranes. This review provides recent studies on PVA-based membranes as alternative anion exchange membranes for alkaline fuel cells. The development of anion exchange membranes in general, including the types, materials, and preparation of anion exchange membranes in the last years, are discussed. The performances and characteristics of recently reported PVA-based membranes are highlighted, including hydroxide conductivity, water uptake, swelling degree, tensile strength, and fuel permeabilities. Finally, some challenging issues and perspectives for the future study of anion exchange membranes are discussed.

## 1. Introduction

The growing population causes problems, including the need for space and the demand for greater resources, which both are limited in number. Fossil fuels as the primary energy source for transportation, industry, and households are running low [1]. In addition, the use of fossil fuels has a negative effect on the environment due to emissions from their combustion. Fossil fuel utilization can release high amounts of carbon dioxide and other greenhouse gases, which can significantly accelerate global warming and climate change, while decreasing air quality. Furthermore, fossil fuels also play a role in sulfur emissions, which cause acid rain, resulting in damage to plants and buildings [2].

Various types of renewable energy continue to be developed as an alternative to fossil fuels. These energies include solar, wind, geothermal, hydropower, biomass, and tidal energy [3]. Although renewable energy has great potential with all its advantages, several issues are still being encountered in its development and use. Among other things, because most renewable energy depends on the climate (e.g., solar, wind, and tidal energy), its generation is not continuous due to variations during the day or in the different seasons. Therefore, its exploitation process requires complex planning, design, and control systems [1,2,3]. Due to the intermittent nature of renewable energy sources, it may open spatial and temporal gaps between the availability of the energy and its consumption by the end-users. In order to address these issues, it is necessary to develop suitable energy storage systems for the power grid [4]. There is also resource competition between food, animal feed, and biomass for energy [5].

Fuel cells are considered one of the potential electricity-producing technologies to meet energy needs in order to overcome the energy crisis. This device generates electricity through an electrochemical reaction between fuel and oxidant. Among the fuels used, hydrogen is the most widely used because of its high efficiency compared to other fuels. Hydrogen as an energy carrier can be attained from many different resources, including fossil fuel, biomass, and water) [1]. This fuel also can be produced with various technologies, such as steam reforming, gasification, chemical looping, and water electrolysis [6]. One of the profitable ways to produce hydrogen gas is by utilizing the surplus electricity generated by renewable energy, such as solar and wind power. Subsequently, we can store the hydrogen to be used later. When electricity production from solar or wind energy is low, fuel cells and stored hydrogen are used to generate electricity [7].

Among the types of fuel cells that have been developed, proton exchange membrane fuel cells (PEMFCs) are the most mature and extensively used fuel cells on account of their high efficiency and power density, as well as low operating temperature, resulting in relatively fast start-up and environmentally safe operation. PEMFCs have high efficiency and power density because the size of the proton (10^−15^ m), which is transported by a proton exchange membrane (PEM), is much smaller than the hydroxide ion (10^−10^ m). Hence it is much easier for PEM to transport protons than an anion exchange membrane (AEM) to transport hydroxide ions [8]. However, its commercialization has been hampered by the dependence on the expensive and limited availability of platinum group metals (PGM) as electrode catalysts, complicated water management, and undesired gas passage through the membrane (fuel crossover) [9,10,11]. The membrane should act as an efficient barrier layer for the active species/fuel in fuel cells. Thus inorganic–organic hybrid PEMs are typically prepared to improve the proton/fuel selectivity and reduce the fuel crossover, which increases both the cost and complexity [8].

**Figure 1:**
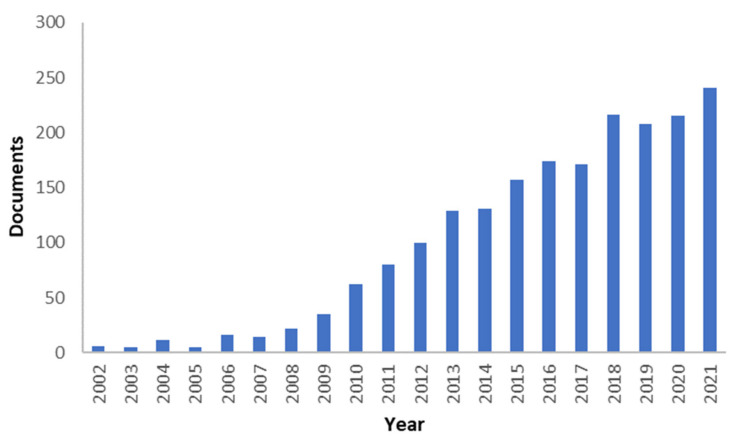
The number of research articles published about AEMFC. Source: https://www.webofscience.com/, accessed on 4 July 2022, keywords: “Anion Exchange Membrane” Fuel Cell (Topic) or “Alkaline Fuel Cell” (Topic) and “Fuel Cell” (Abstract).

Over the past few years, anion exchange membrane fuel cells (AEMFCs) has acquired greater attention among all distinct types of fuel cells. This is due to the fact that AEMFCs have several advantages compared to the proton exchange membrane fuel cell. The benefits of AEMFCs include the possibility of employing non-platinum group metals (non-PGM) catalyst, a high oxygen reduction reaction (ORR) rate, good corrosion resistance of commonly used cell materials in an alkaline environment, and low fuel crossover owing to reverse direction between fuel and hydroxide ion [12]. Figure 1 shows the number of peer-reviewed articles on AEMFC that have been published in the last two decades. There has been a rapid increase from year to year, especially in the last decade.

Anion exchange membrane (AEM), as an anion-conducting polymer electrolyte, is one of the critical components of AEMFCs due to its crucial role in its functionality of AEMFCs [12]. There are several issues in AEM development. Generally, an anion exchange membrane possesses a lower ion conductivity compared to a proton exchange membrane because the mobility of hydroxide ion (OH^−^) is intrinsically inferior to the mobility of proton (H^+^) [13]. The cation group in AEM also shows a tendency to be easily degraded or unstable in a strongly alkaline environment [14]. Some polymers used as backbone materials require high temperatures to dissolve, relatively expensive and ecologically harmful inorganic solvents, and complex synthesis routes, resulting in AEM production being costly, complicated, and less eco-friendly [15]. Therefore, the development of applicable anion exchange membranes that possess a high OH^−^ conductivity, exceptional mechanical strength, and a prolonged period of chemical stability has grown to be one of the foremost challenges in developing AEMFCs [12].

In order to achieve the expected performance of AEMs for AEMFCs, to date, various types of AEMs synthesis approaches have been pursued. The development of AEMS includes the selection of new materials for the polymer backbone [13,16,17,18,19], functionalization of the polymer backbone with alternative functional groups or cations as hydroxide conductors [10,20,21,22,23,24,25,26,27,28], designing membranes with various structures [29,30,31,32], using different techniques or membrane manufacturing methods for processing polymer solution [22,33,34,35], and incorporating various additives to improve membrane performances [36,37,38].

This review provides recent studies on PVA-based membranes as alternative anion exchange membranes for alkaline fuel cells. The development of anion exchange membrane fuel cells and anion exchange membranes in general, including the types, materials, and preparation of anion exchange membranes in the last years, are summarized. The performances and characteristics of recently reported PVA-based membranes are emphasized, including hydroxide conductivity, water uptake, swelling degree, tensile strength, and fuel permeabilities. Lastly, some challenging issues and perspectives for the future study of anion exchange membranes are considered.

## 2. Fuel Cell

A fuel cell is a device that continuously converts the chemical energy in fuels into electricity by means of electrochemical reactions. A typical fuel cell consists of a cathode (positive electrode), an anode (negative electrode), and an electrolyte where ions are transferred from one electrode to another. A fuel and an oxidizing agent (often oxygen) are then supplied to the anode and cathode, respectively.

**Figure 2:**
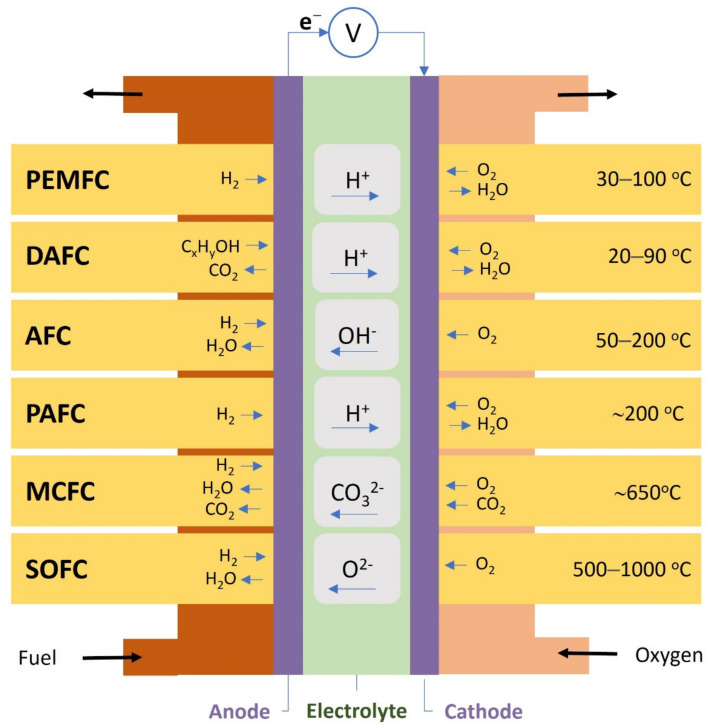
Classification of Fuel Cells (range temperature data adapted from [39]).

Based on the type of electrolyte or fuel, fuel cells are generally categorized into proton exchange membrane fuel cell (PEMFC), direct alcohol fuel cell (DAFC) (using methanol or ethanol as a fuel), alkaline fuel cell (AFC), phosphoric acid fuel cell (PAFC), molten carbonate fuel cell (MCFC), and solid oxide fuel cell (SOFC) (Figure 2).

The proton exchange membrane fuel cell (PEMFC) is the most widely used type of fuel cell. A large power density, eco-friendliness, quick start-up, and diverse possibilities for application are some of the advantages of this technology [40,41]. Even though the proton exchange membrane fuel cell (PEMFC) has substantial performance and promising prospects, several issues have hindered its commercialization. These issues include high cost owing to an expensive platinum catalyst, sluggish reaction kinetics in acidic conditions (particularly the oxygen reduction reaction), a complex water system, and fuel crossover [9,10].

Classical alkaline fuel cell (AFC), which uses alkali electrolyte solution, e.g., KOH and NaOH, offer some technical advantages over PEMFC. AFC has faster ORR kinetics and lower activation overpotential than acidic fuel cells. AFC can operate with non-PGM catalysts and have good ORR kinetics as well as corrosion resistance. However, AFC has a bulky structure and fuel crossover as issues. Liquid electrolytes have a susceptibility to leakage, and electrolytes are poisoned by CO_2_ if no countermeasures are taken. These issues become a major drawback and lead to the interest in this type of fuel cell lessening [42,43].

## 3. Anion Exchange Membrane Fuel Cell

Recently, anion exchange membrane fuel cell (AEMFC) received more attention among all different types. AEMFC has several advantages compared to the proton exchange membrane fuel cell (PEMFC), including the possibility of employing non-platinum group metals (non-PGM) catalyst (e.g., silver and nickel), high oxygen reduction rate (ORR), good corrosion resistance in an alkaline environment, and low fuel crossover owing to reverse direction between fuel and hydroxide ion [12].

Anion exchange membrane fuel cell (AEMFC) is an alkaline fuel cell that employs an anion exchange membrane instead of aqueous potassium hydroxide (KOH) as an electrolyte to conduct hydroxide between the anode and cathode compartments. AEMFC is also known as alkaline polymer electrolyte fuel cell (APEFC), alkaline membrane fuel cell (AMFC), solid alkaline fuel cell (SAFC), and hydroxide exchange membrane fuel cell (HEMFC) [44,45,46].

**Figure 3:**
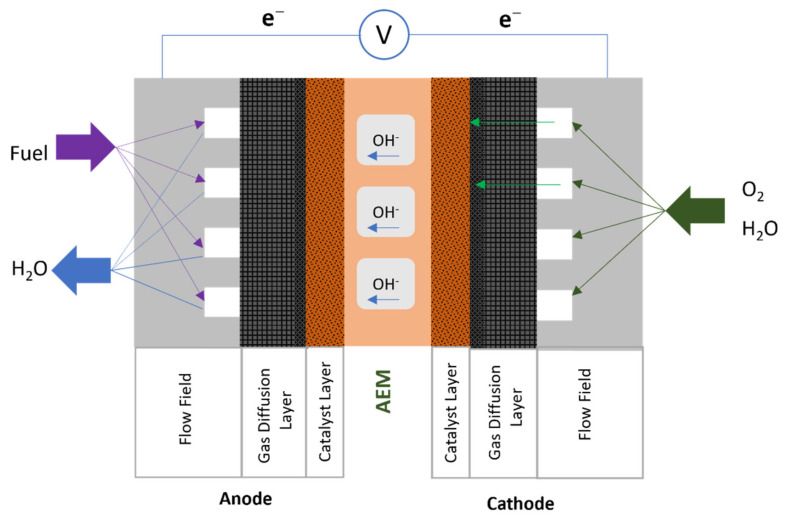
Schematic diagram of AEMFC.

In an AEMFC (Figure 3), the fuel (e.g., hydrogen, methanol, or ethanol) is supplied at the anode, while oxygen and water are provided at the cathode. At the cathode catalyst layer, oxygen is reduced to generate hydroxide ions (OH^−^), which are then transferred through the AEM towards the anode. The fuel (e.g., hydrogen) undergoes oxidation when it reacts with hydroxide ions, producing water and electrons at the anode catalyst layer. The electrons flow through the external circuit to generate current. The reactions that occur in the fuel cell with hydrogen, methanol, and ethanol fuels are shown in Table 1.

## 4. Anion Exchange Membrane

An anion exchange membrane (AEM) is a semipermeable or selectively permeable membrane with positively charged functional groups and a role in conducting anions but rejects cations or some gases. AEMs are widely used in a wide range of applications which are broadly divided into two main groups, namely water-based and energy-based applications [48]. The water-based applications primarily include diffusion dialysis, electrodialysis, and membrane capacitive deionization. On the other hand, several energy-based applications include reverse electrodialysis, redox flow battery, water electrolysis, and fuel cells [48,49].

**Figure 4:**
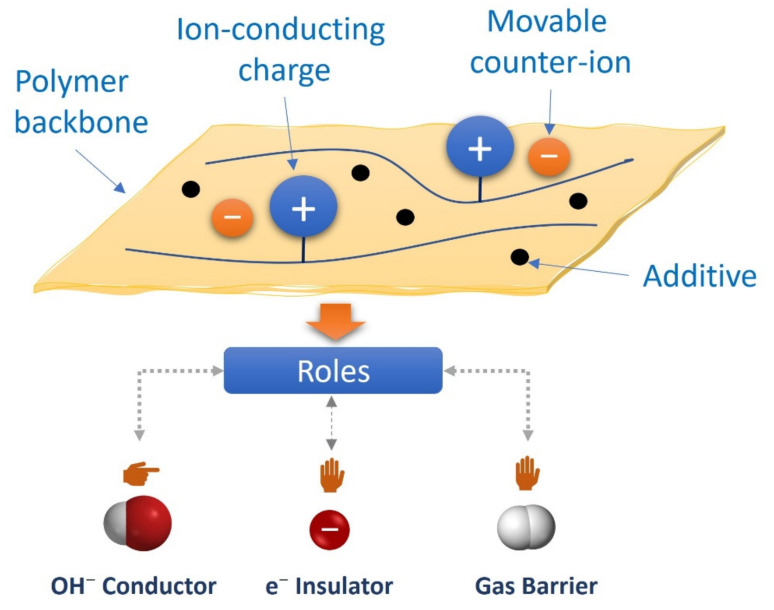
Schematic of Anion Exchange Membrane Structure.

Anion exchange membranes (AEMs) are one of the core components of alkaline fuel cells due to their critical role as ion conductors, electron insulators, and gas barriers. They are composed of a polymer backbone, immobilized cation-functionalized charge groups for conducting OH^−^, movable counter-ions, and additives [49]. The schematic of the membrane structure can be seen in Figure 4.

In order to be used in alkaline fuel cells, an anion exchange membrane should fulfil several requirements, including (1) high OH^−^ conductivity, (2) chemically and thermally stable cationic moieties and polymer backbones, (3) exceptional mechanical properties, (4) sufficient hydration level to facilitate hydroxide transport, and (5) favorable selective permeability to conduct ions while impeding the crossover of fuel and/or oxygen [14,32].

### 4.1. Types of Anion Exchange Membrane

**Figure 5:**
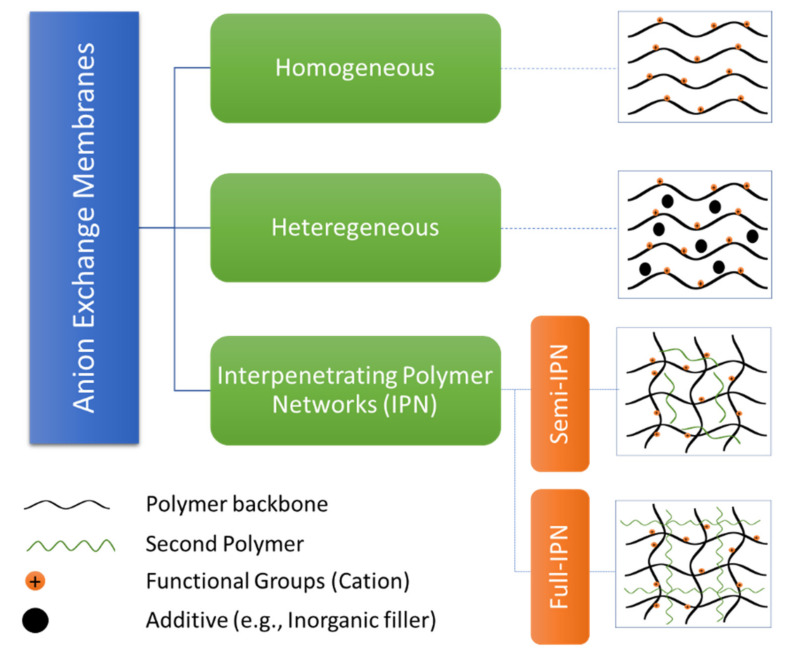
Anion Exchange Membrane types.

According to their structures, AEMs can as well be categorized into three groups: homogeneous, heterogeneous, and interpenetrating polymer networks (IPNs), as it is presented in Figure 5.

#### 4.1.1. Homogeneous Anion Exchange Membrane

Homogeneous AEMs are composed of an ion-exchanging material with a single-phase structure, in which the cationic functional charges are covalently bound to the polymer backbone. Preparation methods for these include (i) direct polymerization: polymerization or polycondensation of monomers that contain cationic moieties; (ii) pre-functionalization: casting a polymer solution that already contains cationic moieties into a film; and (iii) post-functionalization: introduction of cationic moieties into a preformed polymer film [29].

#### 4.1.2. Heterogeneous Anion Exchange Membrane

Heterogeneous AEMs are composed of an ion-exchanging material and an inert matrix material. These AEMs can be either an ion-solvating polymer or a hybrid membrane. The first group is composed of casted polymers (such as PEO, PVA, Chitosan, and PBI [31,32]) doped in the conducting ion’s salt solutions (e.g., KOH). Here the polymers only contribute to mechanical properties. Hybrid membranes, on the other hand, are composed of an organic and an inorganic segment, where the organic compound (i.e., ion-exchanging polymer) provides the conducting properties, and the inorganic component, such as alkoxysilane, oxides (e.g., SiO_2_, Al_2_O_3_, TiO_2_, and ZrO_2_), and nano-carbon (e.g., graphene, CNT) are added to enhance the mechanical, thermal, chemical, and ion-conducting properties [15].

In order to incorporate the inorganic material into the polymer matrix, several techniques, including blending, sol-gel, infiltration, intercalation, in situ polymerization, and molecular self-assembly, are used [14]. The most straightforward method is blending or directly mixing inorganic particles into the polymer resin solution. The inorganic particles are prepared prior to membrane preparation. The mixing can be conducted by melt- or solution blending, followed by polymer solidification. Particle aggregation is the main problem encountered in the blending method. The surface modification of inorganic particles is one of the alternative ways to mitigate this issue [50].

Sol-gel is the most common technique for preparing hybrid or composite membranes. Nano-sized inorganic particles are in situ produced inside the matrix of polymers by hydrolysis as well as the condensation reactions of inorganic alkoxide precursors. The solidification process of polymer chains then accompanies this step.

Another method to prepare hybrid membranes is the infiltration method. Hybrid membranes are prepared after membrane formation, and generally, the inorganic particle precursors infiltrate into a swollen or hydrogel-like polymer matrix. The purpose of the swollen form is to increase the pore or void volume and enable infiltration. Subsequently, the inorganic–organic hybrid membranes are obtained through nano-sized particle growth, impurities removal, and polymer curing [50,51].

#### 4.1.3. Interpenetrating Polymer Networks (IPNs)

An interpenetrating polymer networks (IPNs) structure combines two polymers in a network without any covalent bonds between them. Covalent bonds only occur in each polymer, forming a crosslinked structure. If the crosslinked structure occurs only in one polymer, it is called semi-IPN. In comparison, covalent bonds arise in each type of polymer called full-IPN [15,32]. These structures have drawn significant interest due to their well-balanced combination of conducting and mechanical properties. One polymer works for ion transport while the other (e.g., a hydrophobic polymer) provides good thermal, chemical, and mechanical properties [14].

### 4.2. Fabrication of Anion Exchange Membrane

**Figure 6:**
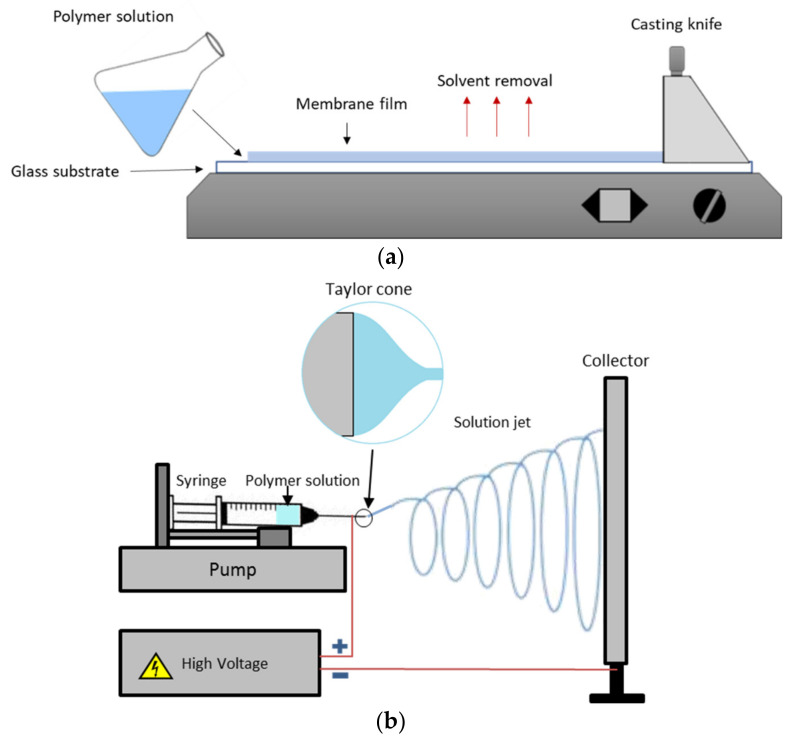
Membrane casting method: (**a**) solution casting; (**b**) electrospinning.

Solution casting (Figure 6a) is arguably one of the most common, versatile, and straightforward techniques for ionomer preparation to form a polymer film or a membrane [33,34,35]. This technique is also called phase separation or phase inversion. Using a casting knife or doctor blade, this method casts the polymer solution to the glass or polytetrafluoroethylene (PTFE) substrate. Subsequently, the polymer film dries under ambient or controlled temperature conditions. Many researchers conducted solution casting with more straightforward laboratory-scale methods, i.e., casting the polymer solution on a petri dish instead of casting equipment.

Electrospinning (Figure 6b) is another method used for AEM preparation for fuel cell application. It can produce a large specific surface area to increase ion exchange capacity. A high voltage creates the electrical force between a liquid polymer drop on a spinneret/needle tip and a collector in the electrospinning process. When the electric field intensity is increased, the surface of the polymer drops on the needle edge lengthens, resulting in a conical shape (i.e., Taylor cone). When the applied electric field reaches a critical value, the repulsive forces exceed the drop’s surface tension, and a solution jet is ejected from the tip of the Taylor cone. Before reaching the collecting screen, the solution jet evaporates or solidifies, and an interconnected web of micro/nanofibers is formed in the collector [52]. The advantage of the electrospinning process is providing the uniaxial alignment of the polymer chains within nanofibers, resulting in enhanced mechanical properties. Another advantage is that electrospinning can stimulate the formation of interconnected channels, which improves ion conduction [52,53,54]. For example, Gong et al. prepared electrospun AEMs using imidazolium-functionalized polysulfone (IMPSF) as material for electrospun nanofibers and fillers to fill the voids between the polymer fibers. Interestingly, when compared to membranes prepared by casting, the OH^−^ conductivity of the IMPSF electrospun AEMs is exceptionally enhanced by 100 times at RH 40% and 1.7 times in water, while the swelling degree is decreased by 35%, and tensile strength increment is up by 22% [22].

### 4.3. Materials for Anion-Exchange Membrane

Polymer backbone materials used in AEMs preparation are classified into aliphatic and aromatic backbones. Poly(vinyl alcohol) (PVA), polytetrafluoroethylene (PTFE), poly(ethylene-co-tetrafluoroethylene) (ETFE), and chitosan (CS) are the most widely studied aliphatic polymer backbones. In contrast, in the aromatic groups, polysulfones (PSU/PSF), polyethersulfone (PES), and poly(2,6-dimethyl-1,4-phenyleneoxide) (PPO) are commonly used. The structures and characteristics of these polymers are summarized in Table 2.

Among ion-functionalized side chains (Table 3), quaternary ammonium (QA) groups have been most intensively studied due to relatively high ion conductivity, reasonable alkaline stability and cost, and the ease of functionalization [55]. However, QA is still unstable in high pH solutions, particularly at high temperatures, because of the degradation through Hofmann elimination (E_2_), nucleophilic substitution (S_N_2), and ylide-formation (Y) [56]. To overcome the limitations of QA in AEMFC application, several functionally charged groups were introduced in AEMs preparation, including imidazolium [57], guanidium [25], phosphonium [58], pyridinium [10,27], and sulfonium [28]. An overview of the structures and common reagents used to introduce these cations are found in Table 3. The most investigated cation after quaternary ammonium is imidazolium since it shows comparable conductivity with QA and low volatility [57]. The favorable imidazolium stability is due to the presence of the p-conjugated imidazole ring, which reduces affinity for S_N_2 substitution and Hofmann elimination [55].

## 5. Poly(Vinyl Alcohol)

Poly(vinyl alcohol) (PVA) is an odorless and tasteless water-soluble polymer. Carbon atoms arrange the backbone of PVA with a hydroxide branch. PVA is semi-crystalline, thermostable, non-toxic, biocompatible, and biodegradable in aerobic and anaerobic environments [80,81,82]. It exhibits remarkable optical properties, exceptional charge storage ability, and large dielectric strength [80].

The vinyl alcohol monomer is unstable, which results in PVA not being prepared by polymerization but by the hydrolysis of polyvinyl acetate. Vinyl acetate monomers are used as a raw material for PVA polymerization in the commercial production process. In this process, the controlled partial hydrolysis of vinyl acetate is carried out in alkaline conditions (i.e., in an environment of aqueous NaOH) where hydroxyl groups partially or almost fully replace the ester groups of vinyl acetate. This reaction produces a precipitate of PVA [80].

**Figure 7:**
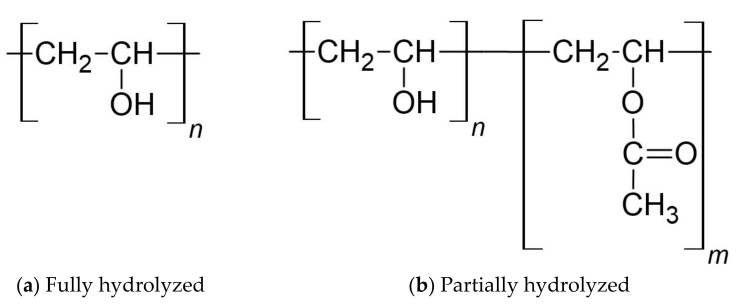
Chemical structures of PVA [81].

The characteristics of PVA are intensely associated with its preparation method, i.e., a full or partial hydrolysis of polyvinyl acetate. Therefore, PVA can be categorized into fully hydrolyzed and partially hydrolyzed (Figure 7). Varying the chain length of poly(vinyl acetate) and the hydrolysis conditions result in poly(vinyl alcohol) products with different properties, including various molecular weights), solubility, adhesiveness, and flexibility. Table 4 summarizes the general physicochemical properties of PVA.

PVA is commercially available in different grades based on the degree of hydrolysis and viscosity. Fully hydrolyzed PVA, which has a hydrolyzation degree of 98% to 99.8%, can be dissolved in water at a temperature of more than 80 °C. On the other hand, partially hydrolyzed PVA with a degree of hydrolysis between 71.5% and 96% has different solubility depending on its molecular weight. The higher molecular weight of PVA requires a higher water temperature for dissolving. Increasing the molecular weight of PVA can also improve the tensile strength, viscosity, specific gravity, crystallinity, and adhesion, whereas flexibility decreases [84].

The utilization and applications of PVA are extensive, including in medical, textile, paper industry, food packaging, construction, water treatment, and energy conversion [80,81,85]. In medical applications, PVA is used for drug carriers, wound dressing, and bone tissue [86]. In textiles, PVA is exploited to produce wool products, hollow yarn fabric, zero twist towels, and so on [87]. PVA is applied in the paper manufacturing process in optical brightener, co-binder, and sizing agent [85]. PVA is employed as a binding and coating agent in the food packaging industry [88]. In construction, PVA is used as a modifier, aggregate, and fiber reinforcing material for cement-based composite materials [89]. PVA also can be used as a biocarrier for water and wastewater treatment in the form of PVA-gel beads [90]. Finally, PVA can be served as an anion exchange membrane material for energy conversion, including water electrolysis and fuel cell [18].

PVA is one of the most widely studied polymers due to its beneficial characteristics for AEM fabrication. The advantages are hydrophilicity which leads to high water uptake, outstanding film-forming capacity, and a high density of reactive chemical groups, which are beneficial for crosslinking by chemical, thermal, or irradiation treatments [91]. The use of PVA-based AEMs is also favorable for mitigating the fuel crossover during the electrochemical process [16]. As a result, the use of PVA as the polymer backbone increased in the last few years. To achieve desired properties, PVA is often combined with other polymers, e.g., chitosan [36], polyethyleneimine (PEI) [92], poly(diallyldimethylammonium chloride) (PDDA) [91], polybenzimidazole (PBI) [93], and polyphenylene oxide (PPO) [94]. In addition, some of the PVA-based membranes also use inorganic compounds, e.g., silica [95], alumina [96], graphene [97], and metal–organic frameworks [94].

## 6. Additives for AEM Preparation

Aside from polymer backbones and cation reagents, in AEMs preparation, several additives are frequently added before or after membrane casting to enhance the membrane’s characteristics. The common additives used in PVA-based AEM preparation are summarized in Table 5.

### 6.1. Crosslinkers

Crosslinking of the polymer chains provides benefits by restraining membrane swelling and improving the membrane’s tensile strength and chemical stability. In many cases, it can reduce the free volume between the main chains due to a network formation and decrease the chain’s mobility and ion-conductivity [108,109]. The crosslinking process can be performed thermally with in situ heat treatment or chemically by adding crosslinking agent additives. It generally involves three types of interactions, i.e., ionic bonding, hydrogen bonding, and covalent bonding. The covalent bond is the most stable and broadly used type for AEMs [110]. Feketefoldi et al. successfully developed a combination of crosslinked quaternized chitosan and quaternized poly (vinyl alcohol) IPN membranes using a combination of two different crosslinking agents (i.e., glutaraldehyde and ethylene glycol diglycidyl ether) and the thermal processes [36]. The membrane with a lower crosslinking degree revealed the best OH^−^ conductivity of 0.016 S cm^−1^ and ion exchange capacity of 1.75 meq g^−1^.

### 6.2. Inorganic Fillers

One way to improve AEM performance is by adding inorganic fillers. Inorganic fillers, including nano-carbons (e.g., CNT, graphene), alkoxysilane, and oxides (e.g., TiO_2_, ZrO_2,_ SiO_2_, and Al_2_O_3_), which are incorporated in the AEM matrix to enhance their thermal, mechanical, chemical and additional electrochemical properties [15]. Movil et al. integrated functionalized graphene oxide (FGO) into polyvinyl alcohol/poly(diallyldimethylammonium chloride) semi-interpenetrating polymer networks (PVA/PDA SIPNs) in AEMs preparation. The results showed that the AEM prepared at a PVA/PDDA weight ratio of 70/30, and 20 wt% FGO has the highest hydroxide conductivity (12.1 mS cm^−1^ at 30 °C and 21 mScm^−1^ at 80 °C) and enhanced thermo-mechanical stability [35].

### 6.3. Plasticizers

Plasticizers can be introduced into the membrane matrix apart from cross-linker and inorganic filler. The plasticizers can improve mechanical properties by decreasing stiffness and thermal stability [111]. A common plasticizer for ion-exchange membrane preparation is poly(ethylene glycol) (PEG). Zhou et al. incorporated poly(ethylene glycol) (PEG) to poly(vinyl alcohol)/poly(acrylamide-co-diallyldimethylammonium chloride) AEMs. The addition of PEG improves the thermal degradation temperature in thermal stability. Water uptake and the conductivity of AEMs also increase with the addition of PEG. The increased WU is due to the strong hydrophilic characteristic of PEG, leading to more water being absorbed in polymer chains. The special plasticizer effect of PEG probably enhanced the mobility of hydroxide charge carriers, thus improving the conductivity [112]

### 6.4. Ionic Liquids (ILs)

Ionic liquids are among the recent additives introduced in the fabrication of AEM. ILs are unique chemical compounds that are salts consisting of cations and anions in liquid form. These chemicals have favorable characteristics, including high ionic conductivity, non-volatility, excellent chemical and thermal stability, and expansive electrochemical windows [106]. When ILs are introduced to the AEM, they can play a role as the “active sites” in the AEM and hasten the mobility of OH^−^ ions [113]. Chu et al. mixed commercial PVA and 1-ethyl-3-methylimidazolium hydroxide ILs ([Bmim]OH) directly to prepare AEM by solution casting. The result exhibits that a membrane with a ratio of [Bmim]OH/PVA of 2 produces the highest conductivity of 19,6 mScm^−1^ at room temperature. This result is nine and nineteen times higher than the [Bmim]OH/PVA ratio of 1 and 0.5, respectively [114]. Wang et al. prepared an AEM using a combination of PVA and geminal-imidazolium-type ILs ([DimL]OH). The conductivity increased with the addition of ILs. The highest conductivity of 58 mScm^−1^ at 70 °C was obtained from a membrane with a PVA:[DimL][OH] ratio of 1:2.5 [106].

## 7. Characteristics and Performance of PVA-Based Anions Exchange Membranes

Membrane characterization includes comprehensive studies on performance, stability, mechanical, and chemical structure. Performances studies comprise water uptake, swelling degree, ion-exchange capacity, ion conductivity, and fuel permeability. Meanwhile, stability studies involve thermal, chemical, and oxidative stability. An analytical method, such as x-ray diffraction analysis, universal testing machine, and scanning electron microscopy (SEM) are used to characterize the membrane’s physical structure, i.e., crystallographic structures, tensile strength, and morphology. Spectroscopic methods, including nuclear magnetic resonance (^1^H-NMR), and Fourier transform infrared (FTIR), are frequently used to investigate the chemical structure and composition of the membrane (e.g., uniform distribution of head groups, the formation of ion clusters). The details of these characterization methods are described by Pourzare et al. [50]. Table 6 summarizes the common characterization methods for ion exchange membranes.

To date, many PVA-based AEMs have been developed. To achieve the desired performance of AEMs for fuel cell development, PVA as the backbone polymer is often combined with other polymers, added with various additives, and varied routes and techniques in the AEMs fabrication. Table 7 summarizes PVA-based AEMs that have been reported in the last decade. Several notable properties are included in the table.

To achieve performance, resilience, and cost goals, the primary requirements of AEMs for alkaline fuel cell applications are:

### 7.1. High OH^−^ Conductivity

The most critical role of the membranes in AEMFCs is as an ionic transport carrier. In AEMFCS, membranes must permeate hydroxide ions only from the cathode to the anode side. A high ionic conductivity (≥100 mScm^−1^) represents a decisive parameter for the membrane in the fuel cell to facilitate large currents with low resistive losses [32]. In PEFCs, the hydrogen ion (or “proton”) carries the electric charge from anode to cathode. In AEMFCs, the hydroxide ion moves from cathode to anode.

**Figure 8:**
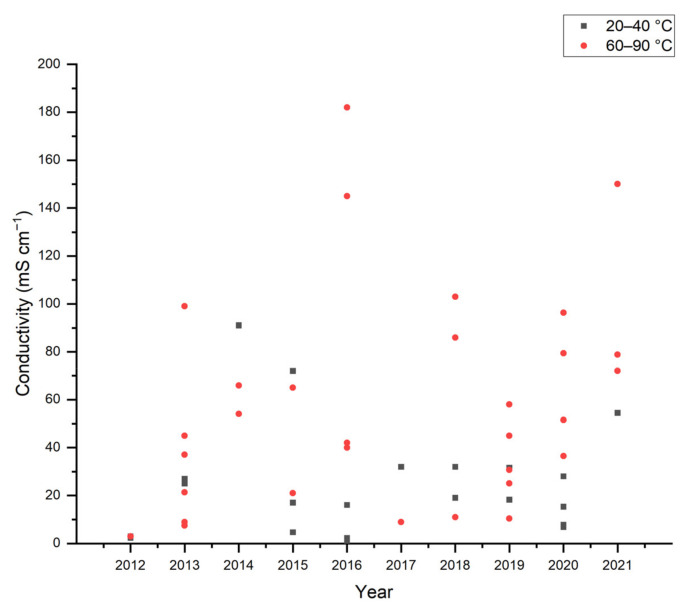
Hydroxide conductivity of PVA-based AEMs [33,34,35,44,59,62,91,92,93,94,95,97,106,112,115,116,117,118,119,120,121,122,123,124,125,126,127,128,129,130,131,132,133,134,135,136,137,139,140,141,142,143,144,145,146,147,148,149,150,151,152,153,154,155,156,157,158].

Figure 8 shows the hydroxide conductivity obtained over the past decade. Since 2013, membranes conductivity has reached values above 100 mScm^−1^ and overcame the primary constraint of AEMs’ low conductivity compared to PEM. In 2016, Wu et al. succeeded in reaching 145 mScm^−1^ of OH^−^ conductivity at 80 °C by entrapping cationic metal–organic frameworks (MOFs) in porous BPPO membranes with PVA acting as a coating on both sides [94]. The cationic MOFs act as OH^−^ conductive passages, whereas the PVA coating can prevent the fuel crossover. Instead of ammonium, Herranz et al. used imidazolium from poly(benzimidazole) (PBI) as a functional group. With a PVA: PBI ratio of 8:1, the ionic conductivity reaches 103 mScm^−1^ at 90 °C, measured with EIS [93]. Gong et al. succeeded in synthesizing full-SIPNs AEM based on quaternary ammonium polysulfone and PVA. The obtained conductivity reaches 180 mScm^−1^ at a temperature of 60 °C with a moderate ion exchange capacity (IEC) of 1.13 meq g^−1^ [130].

The OH^−^ is transported through the AEMs along a water molecule chain by forming and breaking the hydrogen bond according to the Grotthuss mechanism [32]. Therefore, the water content in AEMs affects ion conductivity and fuel cell performance strongly. Parameters that describe water content in AEMs are water uptake (WU) and swelling degree (SD). Water uptake refers to weight change, while the swelling degree defines the dimensional stability of membrane after contact with water for a particular time. The challenge in developing AEMs is how to acquire high WU values that promote high ion conductivity with low SD that indicates good dimensional stability.

**Figure 9:**
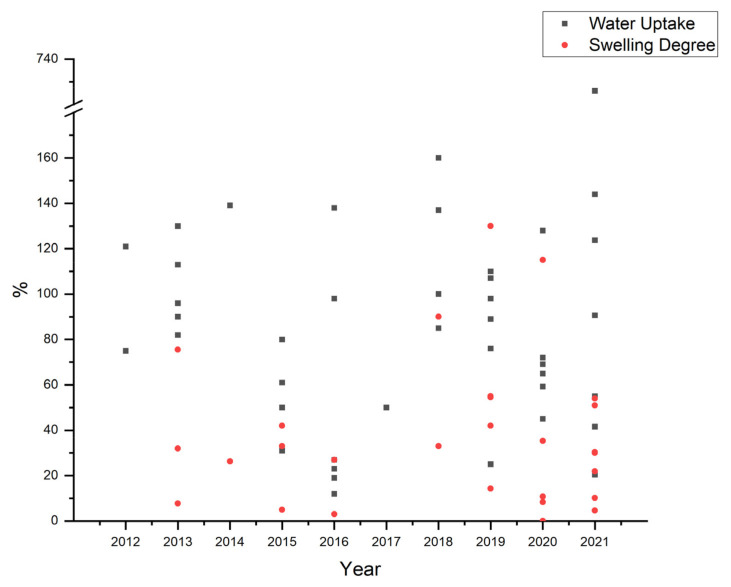
Water uptake and swelling degree of PVA-based AEMs [33,34,35,44,59,62,91,92,93,94,95,97,106,112,115,116,117,118,119,120,121,122,123,124,125,126,127,128,129,130,131,132,133,134,135,136,137,139,140,141,142,143,144,145,146,147,148,149,150,151,152,153,154,155,156,157,158].

Figure 9 depicts the water uptake and the swelling degree of several AEMs. Zhou et al. prepared PVA/Quaterized hydroxyethylcellulose ethoxylate (QHECE) with the solution casting method and compared WU and SD between the produced membrane and Nafion 115 [122]. PVA/QHECE has equivalent WU seven times lower SD than Nafion 115. However, the conductivity is still very low compared to Nafion 115 [120]. Lu et al. fabricated cellulose nanocrystal (CNCs)-based composite AEMs using a combination of PVA and silica gel as a hydrophobic binder. The results showed a low SD of 5% and high WU of 80%, owing to the hydrophilicity and the exceptional dimensional stability of cellulose nanocrystal [127]. Yuan et al. performed the acetal functionalization of imidazole-4-carbaldehyde on multication crosslinked PVA hydrogel membranes. This method produces AEMs with high ionic conductivity of 150 mS with a very high water uptake of 726% and a swelling degree of 54%. However, the mechanical properties of this membrane are not sufficient, and it only reaches a tensile strength of 1.4 MPa [151]. Ari et al. prepared composite AEMs with polycarbonate using imidazolium and quaternary ammonium as anion conducting charge. Polycarbonate can help reduce swelling degrees by 45% less than without polycarbonate [146]. Gong et al. compared the preparation method for IMPSF-based AEMs between electrospinning and solution casting methods. Although water uptake is higher, the electrospun AEMs show a lower swelling degree than cast AEMs. Interestingly, when compared to membranes prepared by casting, the water uptake of the electrospun AEMs is higher by 1.7 times, while the swelling degree is lower by 38% [22]. It is reported that the absorbed water molecules are prone to aggregate along fiber surfaces instead of distributing uniformly in the membrane, which promotes dimensional stability under hydration. The interconnected fiber networks and exceptional interfacial compatibility between the fiber and matrix could also contribute to the excellent swelling resist behavior of the electrospun AEMs [22].

### 7.2. Excellent Mechanical and Thermal Stability

Mechanical stability is one of the crucial factors for AEMs that affect fuel cell lifetime. Nevertheless, AEMs, which are mainly hydrocarbon-based, are prone to be mechanically weaker than the PEMs that that dominated by perfluorinated polymers. Membranes for alkaline fuel cells are expected to be as thin as possible (<50 µm) to lower the resistivity and reduce the system costs [32]. At operating conditions, fuel cells undertake deviations in humidity that cause water sorption and desorption in the membrane. The repeated swelling and contraction of the membrane produce significant mechanical stresses in the membrane. These hygrothermal phenomenon effects lead to pinhole and crack formations that deteriorate the membrane, triggering the subsequent mechanical failure of the membrane [159].

**Figure 10:**
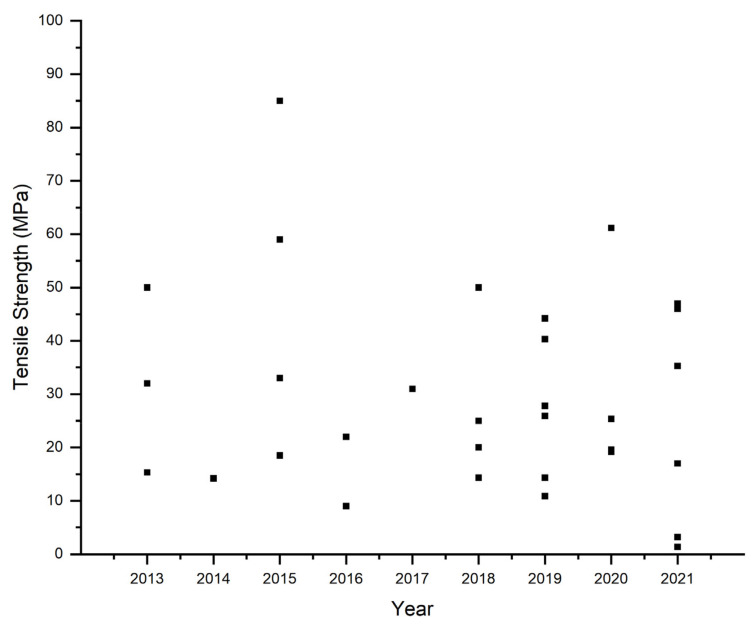
Tensile strength of PVA-based AEMs [34,62,92,93,94,97,106,112,117,123,124,125,126,127,131,135,136,137,140,141,142,143,147,148,149,150,152,154].

Figure 10 shows the tensile strength of PVA-based AEMs. Ye et al. reported thoroughly exfoliated graphene nanosheets and PVA-based manufactured by a blending method [97]. The tensile strength of membranes increased with the addition of graphene. The introduction of 1.4% graphene increased the membrane’s tensile strength, i.e., 72.9% and 13.3% in a wet state. Yang et al. prepared crosslinked poly(vinyl alcohol)/polyquaternium-10 (PVA/PQ-10) membranes with SIPN structure using solution casting. The best mechanical properties of the membranes were achieved with an equal mass ratio of PVA/PQ-10 and glutaraldehyde mass content of 3%, which tensile strength of 61,18 MPa [142]. Sharma et al. synthesized PVA/quaternized polyethyleneimine (PEI) with the addition of functionalized graphene oxide (f-GO). The incorporation of f-GO increases the tensile strength and conductivity compared to those without f-GO up to 200% and 56%, respectively. These results make this type of membrane the highest tensile strength of any PVA-based membrane reported (i.e., 85 MPa) [126].

### 7.3. Electron Insulator and Reactant Barrier

The other function of the membranes in AEMFCs is to be a barrier for anode and cathode reactants, as well as electrons (electronic insulators). AEMs in AEMFCs are designed only to transport the ions, but some electrons and gases (fuel and air) pass through the membranes. These electrons are lost and cannot be used in the external electric circuit. Similarly, an amount of fuel and air diffuses through the membrane and reacts chemically on the opposite electrode to produce heat without generating any electric current. These phenomena are called fuel and oxygen (air) crossovers. Internal currents and gas crossover in fuel cells reduce the current density and efficiency [160].

**Figure 11:**
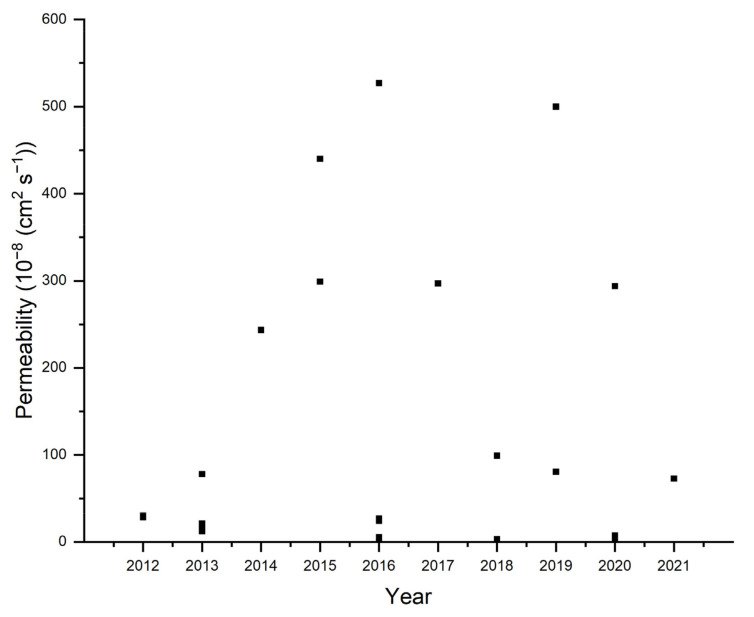
Alcohol Permeability of PVA-based AEMs [62,94,97,106,112,115,118,119,122,126,128,129,131,132,133,134,137,138,143,144,146,150,153,156].

The permeability of PVA-based AEMs for non-hydrogen fuels (i.e., ethanol or methanol) is presented in Figure 11. Jiang et al., achieved permeability of 3 × 10^−8^ cm^2^ s^−1^ by adding 0.5% molybdenum disulfide (MoS_2_). The MoS_2_ nanosheets in ionic channels apparently could increase the tortuosity of the membrane and could effectively hinder methanol transportation through the membrane [62]. Feketefoldi et al. synthesized a series of quaternized chitosan and quaternized poly (vinyl alcohol) membranes using different amounts of glutaraldehyde and ethylene glycol diglycidyl ether as crosslinkers. The ethanol permeability decreases with an increasing degree of crosslinking of the membranes and rises with increasing temperature [36]. Ari et al. reported that imidazolium functionalized AEMs show lower methanol permeability than quaternary ammonium functionalized AEMs. This is due to the lower affinity of imidazolium to water and methanol compared to quaternary ammonium. In addition, the presence of the imidazole ring can inhibit the interaction with the methanol molecule, thereby reducing the permeability. The best permeability achieved is 3 × 10^−8^ cm^2^ s^−1^, which is the lowest permeability so far achieved [146].

### 7.4. Fabrication Cost

Among the challenges faced in developing fuel cells is the high price of commercial membranes. However, many researchers have developed materials and membrane manufacturing techniques that are more economical, with the performance close to or even exceeding the commercial membrane. According to Advanced Research Projects Agency-Energy (ARPHA-E), the cost for AEMs that can be practically integrated into a fuel cell device is less than 20 USD/m^2^ [161]. Table 8 summarizes several commercial membranes used for fuel cells. From Table 8, we can see that the average conductivity for AEMs is still much lower when compared to PEMs/CEMs. Furthermore, membrane prices are still high for both AEMs and CEMS, still far from the applicable target cost according to ARPHA-E.

## 8. Summary and Perspectives

This review discusses and summarizes the development of anion exchange membranes in general (AEMs) and PVA-based AEMs in particular. The types, materials, and preparation of anion exchange membranes in the last years are reviewed. Furthermore, the characteristics of PVA and the performances of recently reported PVA-based AEMs are also reviewed, including hydroxide conductivity, water uptake and swelling degree, tensile strength, and fuel permeabilities.

PVA-based AEMs have potential for alkaline fuel cell applications. Among the noteworthy advantages of PVA are the large number of reactive groups that allow it to be chemically modified, as well as compatibility with other polymers, ion-conducting cations, and additives. Thus, many approaches are available to improve the physicochemical and electrochemical properties of PVA-based AEM. In the last few years, the development of PVA-based AEM resulted in a significant increase in conductivity values approaching 200 mScm^−1^ and showing lower fuel permeability. The mechanical properties of PVA-based membranes show satisfactory results.

Although PVA-based AEMs show potential for alkaline fuel cell application, some challenges should be further investigated to ensure the performance of the membrane has been continuously improved. In order for PVA-based membranes to promote better performance in alkaline fuel cells, the hydroxide conductivity must be further enhanced. This improvement can be the selection of new materials, modification of cation functional groups, development of membrane structures, use of conductive additives, etc. It is also essential to improve the mechanical properties of the membrane, especially in wet conditions. Among these possibilities is the use of a better crosslinker, which can reduce the swelling properties of the membrane while maintaining its high water uptake. The introduction of functionalized inorganic filler is an alternative to improve the mechanical properties as well as the conductivity of the membrane. Since alkaline full cells should be operated at alkaline conditions for an extended period, the chemical stability of the membrane becomes crucial. More efforts should be devoted to improving the stability of the AEMs.

## Figures and Tables

**Table 1:** Reaction in fuel cells (complete oxidation) according to fuel types (Adapted from [47]).

	Fuel Types
	Hydrogen
Anode:	2H_2_ + 4OH^−^ → 4H_2_O + 4e^−^
Cathode:	O_2_ + 2H_2_O + 4e^−^ → 4OH^−^
Cell reaction:	H_2_ + O_2_ → 2H_2_O
	Methanol
Anode:	CH_3_OH + 6OH^−^ → CO_2_ + 5H_2_O + 6e^−^
Cathode:	3/2O_2_ + 3H_2_O + 6e^−^ → 6OH^−^
Cell reaction:	CH_3_OH + 3/2O_2_ → CO_2_ + 5H_2_O
	Ethanol
Anode:	CH_3_CH_2_OH + 12OH^−^ → 2CO_2_ + 9H_2_O + 12e^−^
Cathode:	3O_2_ + 6H_2_O + 12e^−^ → 12OH^−^
Cell reaction:	CH_3_CH_2_OH + 3O_2_ →2CO_2_ + 3H_2_O

**Table 2:** Common polymer backbone materials for AEM.

Polymers	Synonyms	Structure	Characteristics	Challenges	Ref
**Aliphatic backbones**					
Poly(vinyl alcohol) (PVA)		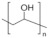	Easy fabrication, biodegradability, hydrophilic, and good chemical stability.	Poor mechanical strength in wet state.	[59,60,61,62]
Polytetrafluoroethylene (PTFE)	Teflon	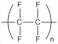	Excellent chemical and thermal stability, low water uptake, and non-toxicity.	Limited synthetic route (impregnation of commercial PTFE film)	[63]
Poly(ethylene-co-tetrafluoroethylene) (ETFE)		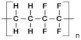	Good mechanical strength, thermal, chemical stability, superior radiation resistance, and feasibility for graft polymerization.	Complex synthetic route, require high-energy electron beam for irradiation.	[64,65]
Chitosan (CS)		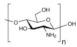	Good film-forming characteristics, good mechanical strength, chemical resistance, low manufacturing cost, biocompatibility, biodegradability, and non-toxicity.	Low conductivity in pristine state	[66,67,68]
**Aromatic backbones**					
Polysulfones (PSU/PSF)	Poly(arylene ether sulfone)	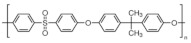	Excellent mechanical and thermal stability, hydrolysis resistance, and wide temperature operating range.	Involves toxic chemicals in the synthesis	[60,69,70]
Poly(ether sulfone)(PES)	Poly(phenylene ether sulfone)	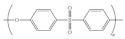	Excellent solubility in an organic solvent, good thermal stability, good mechanical properties, and chemical resistance.	Using organic solvents which are mostly toxic and expensive	[71,72,73]
Poly(ether ether ketone) (PEEK)		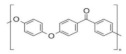	Good mechanical properties, good chemical and thermo-oxidative stability, and low production cost.	Involves toxic chemicals in the synthesis	[74,75,76]
Poly(2,6-dimethyl-1,4-phenyleneoxide) (PPO)	Poly(phenylene ether) (PPE)	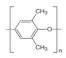	Excellent mechanical properties, good dimensional stability, low moisture uptake, high thermal stability, low flammability, low dielectric constant, and low dielectric dissipation factor.	Involve carcinogen chloromethylation reagents, complex synthesis without nontoxic reagent	[60,77,78,79]

**Table 3:** Common functional charge groups for AEM.

Cations	Structure	Cation Reagents
Quaternary Ammonium	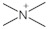	Trialkylamine includes trimethylamine [20], triethylamine, and tripropylamine [21]
Imidazolium	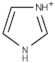	1-methylimidazole [22], 1,2-dimethylimidazole) [23]
Guanidinium	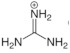	1,1,2,3,3-pentamethylguanidine (PMG) [24] Guanidinium hydrochloride [25]
Phosphonium	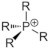	Tris(2,4,6-tri-methoxyphenyl) phosphine (TTMPP) [26]
Pyridinium	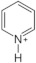	2,6- Bis(4-hydroxyphenyl)pyridine [27] 4-vinyl pyridine [10]
Sulfonium	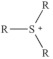	Dimethyl sulfide sulfide [28]

**Table 4:** General Physical and Chemical Properties of PVA.

Parameters	Description
Molecular weight	30,000 to 200,000 g/mol [80]
Physical appearance	white to cream-colored, odorless, powder or granular [80]
Solubility	Soluble in water. Insoluble in oils, ketones, ester, aromatic and aliphatic hydrocarbons [80]
Density	1.19–1.31 g/cm^3^ [80]
Melting point	180–230 °C [83]
Thermal properties	Discoloration: ~150 °C [80] Darken: >150 °C [80] Decomposition: ~200 °C [80]
Viscosity	2.5–110.0 mPa.s [83]
Glass transition temperature	75–85 °C [80]
Structural formula	
Fully hydrolyzed	(-CH_2_CHOH-)-*_n_*-
Partially hydrolyzed	(-CH_2_CHOH-)-*_n_*-(-CH_2_CHOCOCH_3_-)-*_m_*- [80]
Empirical formula	
Fully hydrolyzed	(C_2_H_4_O)*_n_*
Partially hydrolyzed	(C_2_H_4_O)*_n_*(C_4_H_6_O_2_)*_m_* [80]
Degree of hydrolysis	
Fully hydrolyzed	98.0–99.8% [80]
Partially hydrolyzed	71.5–96.0% [80]

**Table 5:** Common additives for PVA-based AEM.

Additives	Objectives	Examples	Ref.
Cross-linker	Restraining membrane swelling and improving the membrane’s tensile strength and chemical stability	- Thermal: annealing - Chemical: dialdehyde (glutaraldehyde, glyoxal), trisodium trimetaphosphate, sodium hexametaphosphate, citric acid, dianhydride, sulfosuccinic acid	[98,99,100,101,102]
Inorganic filler	Enhance the thermal, mechanical, chemical, or additional electrochemical properties	- Nano-carbons: CNT, GO - Oxides: TiO_2_, ZrO_2,_ SiO_2_, and Al_2_O_3_ - Alkoxysilane	[37,96,103,104,105]
Plasticizer	Improve mechanical properties by decreasing stiffness and thermal stability	- Poly(ethylene glycol)	[38]
Ionic liquids (ILs)	Increase conductivity of AEM	- Geminal-imidazolium-type ILs - 1-ethyl-3-methylimidazolium ILs	[106,107]

**Table 6:** Characterization method for ion-exchange membrane.

Properties	Unit	Description/Purposes	Method
		**Performances**	
Ion exchange capacity (IEC)	mmol g^−1^ or meq g^−1^	Implies the milli-equivalents number of exchangeable ions in 1 g of the dry membrane	Back-titration
Ion conductivity		Measure the proton conductivity of PEMs or OH^−^ conductivity of AEMs.	Electrochemical impedance spectroscopy (EIS)
Water uptake (WU)	% or g/g	Investigate the changes in membrane mass when exposed to water	Gravimetric
Swelling degree (SD)	% or g/g	Investigate the dimensional change of the membranes when exposed to water	Length measurement
Fuel permeability	cm^2^ s^−1^	Investigate fuel crossover by diffusion due to the concentration gradient and by the electroosmotic drag as well.	Side-by-side cell
Thermal stability	% (weight)	Investigate the change in the weight of membrane temperature over a certain period.	Thermogravimetric analysis (TGA)
Chemical stability	% (conductivity)	Investigate the AEMs performance changes (ionic conductivity and IEC) over time when exposed to high pH environments at a specific temperature	Identical with IEC and ionic conductivity measurement
Oxidative stability	% (weight)	Investigate the oxidative stability of the membrane.	Gravimetric
		**Physical Structure**	
Crystallographic structures	% (crystallinity)	Investigate the crystallographic structure of inorganic materials in the membrane.	X-ray diffraction analysis (XRD)
Tensile strength	MPa	Investigate tensile strength of membranes.	Universal Testing machine
Elongation at break	% (length)	Investigate elongation at break of membranes.	Universal Testing machine
Morphology	-	Investigate the surface and cross-section morphology of membranes	Scanning electron microscopy (SEM)
**Chemical structure**			
Polymer structure and chemical composition	-	Investigate changes in chemical structure due to chemical modification	FTIR spectroscopy ^1^H-NMR spectroscopy

**Table 7:** Reported PVA-based AEMs and their notable properties.

Polymers	Cation	Additives *	Preparation	Ion Conductivity	Temp.	Water Uptake	Swelling Degree	Tensile Strength	Alcohol Permeability	Ref.
				(mS cm^−1^)	(°C)	(wt%)	(%)	(MPa)	(cm^2^ s^−1^)	
QPVA	Ammonium	Glutaraldehyde (*CL*), QSiO_2_ (*F*)	Solution Casting	2.4	25	N/A	N/A	N/A	N/A	[95]
PVA/PAADDA	Ammonium	Glutaraldehyde (*CL*)	Solution Casting	3.0	80	75	N/A	N/A	2.85 × 10^−7^	[115]
PVA/PDDA	Ammonium	Glutaraldehyde (*CL*)	Solution Casting	25.0	25	96	N/A	N/A	N/A	[116]
PVA/PDDA	Ammonium	Glutaraldehyde (*CL*)	Solution Casting	27.0	25	130	76	15.3	N/A	[117]
PVA/Cu(II) complex	N/A	Glutaraldehyde (*CL*)	Chemical Fiber	99.0	77	90	32	N/A	2.12 × 10^−7^	[118]
PVA/PDDA	Ammonium	Glutaraldehyde (*CL*)	Solution Casting	37.0	80	96	N/A	N/A	N/A	[91]
PVA/PAADDA	Ammonium	Glutaraldehyde (*CL*), PEG (*P*)	Solution Casting	9.0	80	113	N/A	N/A	N/A	[112]
PVA/QPEI	N/A	Glutaraldehyde (*CL*)	Solution Casting	45.0	80	82	N/A	N/A	7.80 × 10^−7^	[119]
PVA	N/A	Glutaraldehyde (*CL*), Graphene *(F)*	Solution Casting	21.3	80	N/A	N/A	50.0	1.91 × 10^−7^	[97]
PVA/QHECE	Ammonium	Glutaraldehyde (*CL*)	Solution Casting	7.5	90	82	8	32.0	1.26 × 10^−7^	[120]
PVA/QASP/ TAMPFS-PET	Ammonium	Glutaraldehyde (*CL*), SiO_2_ (*F*)	Solution Casting	65.9	70	N/A	N/A	N/A	N/A	[121]
PVA/SA	N/A	Glutaraldehyde (*CL*)	Solution Casting	91.0	25	314	330	N/A	2.43 × 10^−6^	[122]
PVAc/PVBC	Ammonium, Imidazolium	PVAc macromolecul (*CL*)	Solution Casting	54.0	80	139	26	14.2	N/A	[123]
PVA/PMVIC-co-VP	Imidazolium	Glutaraldehyde (*CL*)	Solution Casting	17.0	25	31	N/A	59.0	N/A	[124]
PVA	N/A	CHDMG (*CL*)	Solution Casting	4.7	25	50	N/A	18.5	N/A	[125]
PVA/QPEI	Ammonium	Glutaraldehyde (*CL*), f-GO (*F*)	Solution Casting	72.0	30	61	33	85.0	4 × 10^−7^	[126]
PVA	N/A	CNC (*F*)	Solution Casting	65.0	60	80	5	33.0	N/A	[127]
QPVA/QCS	Ammonium	Glutaraldehyde (*CL*)	Solution Casting	21.0	60	N/A	42	N/A	2.99 × 10^−6^	[128]
PVA/PDDA	Ammonium	Glutaraldehyde (*CL*), f-GO (*F*)	Solution Casting	21.0	80	N/A	N/A	N/A	N/A	[35]
CS/PVA	N/A	Glutaraldehyde (*CL*)	Solution Casting	0.2	25	138	N/A	N/A	2.43 × 10^−7^	[129]
QPVA/QCS	Ammonium	Glutaraldehyde, EGDGE (*CL*)	Solution Casting	16.0	25	98	N/A	N/A	3.17 × 10^−8^	[36]
PVA/BPPO	Ammonium	Glutaraldehyde (*CL*), MoF (*F*)	Solution Casting	145.0	80	27	N/A	22.0	2.68 × 10^−7^	[94]
QPSF/PVA	Ammonium	TMEDA (*CL*)	Solution Casting	182.0	60	12	27	14.0	N/A	[130]
PVA/PDDA	Ammonium	Glutaraldehyde, PEDGE (*CL*)	Solution Casting	2.3	25	N/A	N/A	N/A	N/A	[33]
QPVA	Ammonium	N/A	Electrospinning	42.0	60	23	N/A	9.0	5.27 × 10^−6^	[131]
QPVA	Ammonium	Glutaraldehyde (*CL*), nano-Chitosan (*F*)	Solution Casting	40.0	70	19	3	N/A	5.41 × 10^−8^	[132]
PVA/CoOOH	Ammonium	N/A		32.0	30	N/A	N/A	N/A	2.97 × 10^−6^	[133]
PVA/PUB	Ammonium	Glutaraldehyde (*CL*)	Solution Casting	9.0	80	50	N/A	31.0	N/A	[34]
PVA/PBI	Imidazolium	N/A	Solution Casting	103.0	90	85	N/A	50.0	N/A	[93]
PVA/CS	N/A	Glutaraldehyde (*CL*)	Electrospinning	19.0	25	160	N/A	N/A	9.92 × 10^−7^	[134]
QPVA/CS	Ammonium	Glutaraldehyde (*CL*), MoS_2_ (*F*)	Solution Casting	32.0	25	137	33	25.0	3 × 10^−8^	[62]
PVA/BPEI	N/A	Glutaraldehyde (*CL*)	Solution Casting	86.0	80	100	90	20.0	N/A	[92]
QPVA	Ammonium	Glutaraldehyde (*CL*)	Solution Casting	11.0	80	N/A	N/A	N/A	N/A	[61]
PVA/FDB18C6	Ammonium	N/A	Solution Casting	25.0	70	25	14	14.3	N/A	[135]
PVA/PDDA	Ammonium	Glutaraldehyde (*CL*), ZrO_2_ (*F*)	Solution Casting	31.6	25	89	42	10.9	N/A	[136]
QPVA/KOH	Ammonium	Glutaraldehyde (*CL*)	Solution Casting	30.7	70	76	55	25.9	8.06 × 10^−7^	[137]
PVA/PDDA	Ammonium	Glutaraldehyde (*CL*), MWCNTs (*F*)	Solution Casting	45.0	80	98	N/A	40.3	N/A	[138]
QPVA/KOH	Ammonium	Glutaraldehyde (*CL*)	Solution Casting	18.2	25	N/A	N/A	N/A	N/A	[139]
PVA-FP/[DimL][OH]	Imidazolium	Glutaraldehyde (*CL*)	Solution Casting	58.0	70	107	55	27.8	5 × 10^−6^	[106]
PVA-PY-DLx	Pyridinium	1,4-dichlorobutane (*CL*)	Solution Casting	10.5	70	110	130	44.2	N/A	[140]
QPPONF/PVA	Ammonium	4-chlorobenzaldehyde (*CL*)	Solution Casting	51.5	60	45	11	19.2	N/A	[141]
PVA/PQ-10	Ammonium	Glutaraldehyde (*CL*)	Solution Casting	79.4	80	59	8	61.2	N/A	[142]
Silica/PVA-Py	Ammonium	Glutaraldehyde (*CL*)	Sol-gel	96.3	80	69	35	25.4	7.57 × 10^−8^	[143]
Cu(OH)_2_-PVA-AER	Ammonium	n-Cu(OH)_2_ (*F*)	Solution Casting	28.0	25	N/A	N/A	N/A	2.94 × 10^−6^	[144]
PVA-PVA (modified)	N/A	N/A	Coating	6.9	25	65	N/A	N/A	N/A	[145]
PVA-Im/PC	Imidazolium	Glutaraldehyde (*CL*)	Coating	7.8	20	72	0	N/A	1.10 × 10^−8^	[146]
QPVA/MGMC	Imidazolium	N/A	Solution Casting	15.3	25	N/A	N/A	N/A	N/A	[44]
QPVA/PDDA	Ammonium	Glutaraldehyde (*CL*)	Solution Casting	36.5	60	128	115	19.6	N/A	[147]
BPPO-PVAIm	Imidazolium	N/A	Solution Casting	78.8	80	42	10	47.0	N/A	[148]
QPVA/PDDA	Ammonium	Glutaraldehyde (*CL*)	Solution Casting	54.5	25	55	51	N/A	N/A	[149]
PVA-CoCp	Cobaltocenium	Glutaraldehyde (*CL*)	Solution Casting	72.0	80	20	5	17.0	N/A	[150]
PVA-TFBA-IM-MC	Imidazolium	TFBA (*CL*)	Solution Casting	150.0	80	726	54	1.4	N/A	[151]
QPVA/PDDA	Ammonium	Glutaraldehyde (*CL*)	Solution Casting	82.9	80	91	30	46.0	N/A	[152]
PVA-HH	Ammonium	Glutaraldehyde (*CL*)	Solution Casting	6.2	70	144	30	35.3	7.29 × 10^−7^	[153]
PVA-PQVBC	Ammonium	Divinylbenzene, Glutaraldehyde (*CL*)	Solution Casting	141.9	80	124	22	3.2	N/A	[154]

* *CL* = cross-linker; *F* = filler; *P* = plasticizer.

**Table 8:** Properties and prices of commercial membranes.

Brand	Company	Product	Thickness (μm)	IEC (mmol g^−1^)	σ (mS cm^−1^)	TS (mPa)	Price (USD/m^2^)	Ref.
Anion Exchange Membranes								
Fumasep^®^	Fumatech, Germany	FAA-3-30	26–34	1.67–2.04	>5 (Cl^−^)	25–40	950	[162]
		FAA-3-50	45–55	1.60–2.10 (Cl^−^)	3–8 (Cl^−^)	25–40	1050	[163]
		FAA-3-PK-75	70–80	1.20–1.40 (Cl^−^)	4.5–6.5 (Cl^−^)	30–60	1600	[164]
Sustainion^®^	Dioxide Materials, USA	X37-50	50	N/A	80	N/A	4583	[165]
Cation Exchange Membranes								
Nafion^®^	The Chemours Company, USA	Nafion^®^ 115	125	>0.9	74	32–43	3434	[166,167]
		Nafion^®^ 117	180	>0.9	70	32–43	3770	[167,168]
Aquivion^®^	Solvay, Belgium	E98-05	50	>1	>160	30–40	1667	[169]

## Data Availability

Data presented in this study are available on request from the corresponding author.
